# Evaluation of potential drug- herb interactions among a group of Palestinian patients with chronic diseases

**DOI:** 10.1186/s12906-015-0764-7

**Published:** 2015-07-11

**Authors:** Rowa’ Al-Ramahi, Nidal Jaradat, Ruba Shalalfeh, Sojoud Nasir, Yazan Manasra, Ihab Shalalfeh, Yasmen Esam

**Affiliations:** Department of Pharmacy, Faculty of Medicine and Health Sciences, An-Najah National University, P.O. Box (7), Nablus, Palestine

**Keywords:** Drug, Herb, Interaction, Palestine

## Abstract

**Background:**

The aim of this study was to find the prevalence of potential drug-herb interactions in patients with chronic diseases and identify factors associated with these interactions if present.

**Method:**

The study was a questionnaire based cross-sectional study. It was conducted at a number of governmental primary healthcare centers which include outpatient clinics for chronic diseases between July and November 2013. Patients come to these clinics monthly or bimonthly to receive their medications for their chronic diseases free. The patients in this study were seen at these clinics and their medications were reported from the most recent prescription in their files.

**Results:**

A total of 400 patients agreed to be interviewed, 209 (52.3 %) were females. The most commonly used medications were metformin, insulin, and enalapril. Among the patients, 237 (59.3 %) were using 395 medicinal herbs. The most commonly used herbs were sage, anise and peppermint. In 51 out of the 237 cases (21.5 %) at least one potential drug-herb interaction was found. Male patients were more likely to have potential drug-herb interactions. Patients with potential drug herb interactions were older, having a higher mean number of chronic diseases and medications (*P*-value < 0.05). Only 133 out of 237 (56.1 %) users told their prescribers or pharmacists before using medicinal herbs.

**Conclusion:**

Use of medicinal herbs is a common practice among Palestinian patients attending primary healthcare centers. A substantial proportion failed to disclose to their doctors or pharmacists about herbal products they used, therefore, the physicians and pharmacists are recommended to ask patients about the use of medicinal herbs to avoid any possible negative outcomes. Better counseling and communication between patients and healthcare providers is recommended.

## Background

Herbal medicine is an increasingly common form of complementary and alternative therapy all over the world. Most herbal products are considered dietary supplements and thus are not regulated as medicines. In some countries, herbal remedies are marketed without previous approval of their efficacy and safety by regulatory bodies [1]. Often when patients are asked about their medications, they report only the prescription drugs that they take. It seems common that over-the-counter products, vitamins, and herbal supplements are excluded from what patients typically think of as drugs [2–4], therefore it is important for physicians and pharmacists to investigate a complete medication history including all the drugs, herbals, vitamins and supplements.

In general herbs are safer than pharmaceutical drugs but the concurrent use of herbal and drug combinations may raise the potential of drug- herb interactions [5]. Several studies concerning drug-herb interactions with clinical significance pointed that these interactions may lead to serious clinical consequences and both pharmacokinetic and/or pharmacodynamic mechanisms have been considered to play a role in these interactions [5, 6]. Many documented interactions are from limited and small case reports and clinical observations and the details around the interactions are sparse, however they support that some herbal medications or supplements have potentially harmful side effects as well as adverse interactions with conventional drugs. Such adverse reactions involve all systems, age groups and severity.

The clinical importance of drug-herb interactions depends on many factors associated with the particular herb, drug and patient. Encouraging data support the efficacy of some popular herbal medicinal products, and the potential for benefit appears greater than harm [1, 6]. As a precaution to consumers, herbs should be appropriately labeled to alert consumers to potential interactions when concomitantly used with drugs, and to recommend a consultation with physicians or pharmacists before use.

Previous studies have shown a very common use of herbs among the Palestinians [7–9]. There are limited data about drug-herb interactions in Palestine. It is important to evaluate the appropriate use of herbals if patients use drugs at the same time. Findings can help in developing educational programs to improve doctors’ and pharmacists’ knowledge around drug-herb interactions to ensure they properly counsel patients to avoid improper use of herbals and drugs.

The aim of this study is to find the prevalence of potential drug-herb interactions in patients with chronic diseases in Palestine and identify factors associated with these interactions if present.

## Methodology

The study was a questionnaire based cross-sectional study that was conducted between July and November 2013. It was at a number of governmental primary healthcare centers which include outpatient clinics for chronic diseases. Patients come to these clinics monthly or bimonthly to receive their medications for their chronic diseases free (if they have a governmental health insurance). The patients in this study were seen at these clinics and their medications were obtained from the most recent prescription in their files. All patients with chronic diseases who visited the chronic disease clinics in the selected centers during the study period and were using at least one medication chronically previously were asked to participate in the study. Based on Raosoft sample size calculator (if margin of error = 5 %, confidence level = 95 %, and response distribution = 50 %), the minimum sample size for this study was 400 patients. Convenience sampling technique was used.

Data collection tool was a face to face questionnaire. Verbal consent was obtained from the participant first. The questionnaire included information about sociodemographic characteristics of the patients, medical history, chronic medications, and herbal remedies used. Patients on herbal remedies were asked about the history of herbal use, indications, frequency and if their healthcare providers were aware of this. The medications were documented from the most recent prescription in the patients’ files. The clarity of the questionnaire was assessed in a pilot study. The study protocol was in compliance with the Helsinki Declaration and it was authorized by An-Najah National University Institutional Review Boards (IRB) and Ministry of Health before initiation of this study. Possible interactions between medications and herbal medicines were reviewed using Mosby's Handbook of Herbs and Supplements and Their Therapeutic Uses [10] and Herbs and Natural Supplements-An Evidence Based Guide [11].

Statistical analysis was performed using Statistical Package for Social Sciences (SPSS version 16.0). Mean ± standard deviation was computed for continuous data. Frequencies and percentages were calculated for categorical variables. Kolmogorov-Smirnov test was used for normality testing. Means were compared using independent samples t-test. Categorical variables were compared using Chi-squared and Fisher’s exact tests, as applicable. A *p*-value of less than 0.05 was considered to be statistically significant for all analyses.

## Results

### Socio-demographic characteristics of patients

During the study period, a total of 400 out of 412 patients agreed to be interviewed giving a response rate of 97 %. The average age (± SD) of patients was 48.66 ± 13.69 years, of these 209 (52.3 %) were females, the majority 281 (70.3 %) were living in a village and more than half of the patients 218 (54.5 %) had a school degree.

### Medications used by the patients with chronic diseases

The 400 patients were using 664 medications with a mean of 1.66 ± 0.98. Regarding their chronic diseases, 144 (36.0 %) had diabetes mellitus (type 1 and type 2), 118 (29.5 %) had hypertension, 43 (10.8 %) had a heart disease and the same was the number of patients with arthritis. The most commonly used medications were metformin in 85 cases, insulin in 71 cases, and enalapril in 44 cases. Table 1 shows the top 15 used medications among patients.

**Table 1:** The frequency and percentage of most commonly used medications in patients with chronic diseases (N = 400)

No.	Medication	Frequency	Percentage (%)
1.	Metformin	85	21.3
2.	Insulin	71	17.8
3.	Enalapril	44	11.0
4.	Atrovastatin	35	8.8
5.	Acetylsalicylic acid	32	8.0
6.	Diclofenac	29	7.3
7.	Valsartan	24	6.0
8.	Atenolol	19	4.8
9.	Salbutamol	17	4.3
10.	Hydrochlorothiazide	16	4.0
11.	Furosemide	15	3.8
12.	Amlodipine	13	3.3
13.	Ipratropium	12	3.0
14.	Oxerutinum	12	3.0
15.	Prednisolone	9	2.3

### Herbs used by the patients with chronic diseases

Among the 400 patients, 237 (59.3 %) were using 395 medicinal herbs. The most common herbs used were sage (48 cases), anise (44 cases) and peppermint (42 cases). Table 2 shows the top 15 used herbs among patients.

**Table 2:** The frequency and percentage of most commonly used herbs in patients with chronic diseases (N = 400)

No.	Herb	Frequency	Percentage (%)
1.	Sage (Salvia officinalis)	48	12.0
2.	Anise (Pimpinella anisum)	44	11.0
3.	Peppermint (Mentha piperita)	42	10.5
4.	Thyme (Thymus vulgaris)	27	6.8
5.	Green tea (Camellia sinensis)	27	6.8
6.	Rosemary (Rosmarinus officinalis)	17	4.3
7.	Lupine (Lupinus albus)	15	3.8
8.	Hawthorn (Crataegus monogyna)	13	3.3
9.	Nigella seeds (Nigella sativa)	8	2.0
10.	Ginger (Zingiber officinale)	8	2.0
11.	Garlic (Allium sativum)	8	2.0
12.	Olive oil (Olea europaea)	8	2.0
13.	Fenugreek (Trigonella foenum)	7	1.8
14.	Castor oil (Ricinus communis)	7	1.8
15.	Zucchini (Cucurbita pepo)	6	1.5

The medications and herbs used in these patients were reviewed for potential drug-herb interactions. At least one potential drug-herb interaction was found in 51 out of the 237 cases (21.5 %) who claimed medicinal herb use.

### Knowledge and beliefs about herbs

Among the 400 patients, 220 (55.0 %) said that they believe medicinal herbs are useful for their conditions. When patients were asked if herbs can cause side effects, 286 (71.5 %) claimed that they do not. When users were asked if they have ever suffered from potential side effects that could be due to the use of herbs, 39 (16.5 %) of the patients answered with yes. Users were asked if they have told their prescribers or pharmacists before using medicinal herbs, 133 out of 237 (56.1 %) said yes, the rest 104 (43.9 %) did not tell their healthcare providers about their use of some herbs for medicinal purposes. Only 8 patients were told by their doctors or pharmacists about possible side effects or interactions between their medications and certain herbs. Among users, 91 (38.4 %) of them said the level of living (economic situation) was a factor for herb use. Regarding the frequency of using medicinal herbs, 78 (32.9 %) of users said that they use them daily and 154 (65 %) told that they only use them as needed.

### Factors associated with potential drug-herb interactions

Table 3 shows the factors associated with potential interactions among patients. There was a significant difference in gender, age, number of medications and number of chronic diseases (P < 0.05). Male patients were more likely to have potential drug-herb interactions. Patients with potential drug herb interactions were older and had a higher mean number of chronic diseases and medications.

**Table 3:** Effect of some sociodemographic factors on potential drug-herb interactions

	Potential Interaction	No interaction	P- value
Gender			0.027^a^
Male	30 (58.8 %)	77 (41.4 %)	
Female	21 (41.2 %)	109 (58.6 %)	
Mean age (Years)	53.1 ± 13.3	46.1 ± 13.8	0.001^b^
Mean no. of medications	2.1 ± 1.1	1.4 ± 0.8	<0.001^b^
Mean no. of diseases	1.6 ± 0.8	1.2 ± 0.4	<0.001^b^

a: Chi-square test

^b^: Independent samples t-test

## Discussion

Among the 400 patients, 237 (59.3 %) used at least one medicinal herb. Compared to other studies this is a high percentage and similar to previous studies from our country where 51.9 % of diabetic patients reported taking herbs [7] and 85.7 % of hypertensive patients used at least one type of complementary and alternative medicines. Of the users, 62.1 % reported taking herbs [8]. According to the World Health Organization (WHO), about 70 % of the world population use medicinal herbs as complementary or alternative medicine [12], so the use of herbal remedies is common worldwide.

The herbs used by patients in this study are relatively safe because most herbs used here are also edible plants used in cooking so they are not some unknown plants that might be toxic. However, even these plants can have potential drug-herb interactions. In 21.5 % of the users in this study at least one potential drug-herb interaction was found. In a study on patients using dietary supplements in two American cities, among patients taking supplements (48 %) of Pittsburgh patients and (44 %) of Los Angeles patients had potential drug–dietary supplement interactions of any significance [13]. So it is important to know that even relatively safe and commonly used products as herbs or supplements may have interactions with medications.

Many herbs were used without telling doctors or pharmacists (in 43.9 %), which could increase the risk of interactions and side effects especially in patients with chronic diseases. This practice might be due to low level of awareness about interactions, or patients might be scared of misjudgment, scared of herbals being taken away from them and desperate to solve health problems; all patients should ask their doctors or pharmacists before they use herbs to have appropriate counseling and health care providers should open this topic for discussion with their patients in a non-judgmental manner. This practice was reported in previous studies from our country where 34.2 % of pregnant women and 68.1 % of hypertensive patients did not tell their doctors that they used herbs [8, 9]. In a study by Goldstein et al. [3], 299 hospitalized patients were interviewed. Of the participants, 26.8 % were herbal or dietary supplement consumers, and of the herbal consumers, 59 % stated that their family doctor was aware of the supplement. The hospital medical team was aware of the herbal consumption of 28 % of the herbal consumers and 94 % of the patients had not been asked specifically about herbal consumption by the medical team. It is important for healthcare providers to ask the patients about this point because patients might think this is not important information to disclose.

Most patients (71.5 %) thought herbs did not cause side effects. People mistakenly think that all herbs are safe, because of the fact that they are natural, but this is not true. Concurrent administration of herbs may interfere with the effect of drugs and they may cause side effects similar to chemical drugs, for example liver damage, heart arrhythmia, hypoglycemia and bleeding [14]. Only 8 (2.0 %) patients were told by their doctors or pharmacists about possible side effects or interactions between their medications and certain herbs. When users were asked if they have ever suffered from potential side effects that could be related to the use of herbs, 39 (16.5 %) of the patients answered with yes. For example, many herbs can cause cardiovascular side effects [15]. Our healthcare providers seem to underestimate this problem.

Regarding the frequency of using medicinal herbs, 78 (32.9 %) of users said that they use them daily and 154 (65 %) said that they use them only when needed and this should be considered when medications are prescribed because daily use means higher possibility of interactions if present. The possibility of drug-herb interactions increased in older patients and with increasing number of medications used by patients and number of chronic diseases, and this is expected. These patients require special attention because polypharmacy and old age are both known reasons for higher drug interactions and adverse effects [16].

The first limitation of this study is that the answers reported by the respondents cannot be validated and recall bias is possible, but this cannot be avoided in survey studies. Another limitation is that the study was performed at a group of healthcare centers; therefore it might not be representative to the practice in other cities, villages and camps. Also, the references used for drug herb interactions were not updated. Another important limitation of this study is that these potential interactions were not assessed or followed to determine their clinical significance. However, these results can give baseline data that can be useful in designing and implementing suitable education and performing other related studies.

## Conclusion

Use of medicinal herbs is a common practice among Palestinian patients attending primary healthcare centers. A substantial proportion failed to disclose to their doctors or pharmacists about herbal products used, so the physicians and pharmacists are recommended to ask patients about this to avoid any possible negative outcomes. Some patients had potential drug-herb interactions which they were not informed about. Better counseling and communication between patients and healthcare providers is recommended. Healthcare providers should be aware of the common herbal medicines used and the evidence-based research regarding potential benefits, side effects and drug herb interactions.
